# A mini review on green nanotechnology and its development in biological effects

**DOI:** 10.1007/s00203-023-03467-2

**Published:** 2023-03-22

**Authors:** Salem S. Salem

**Affiliations:** Botany and Microbiology Department, Faculty of Science, AL-Azhar University, Nasr City, Cairo, 11884 Egypt

**Keywords:** Green nanotechnology, Biological synthesis, Nanoparticles, Application

## Abstract

The utilization of living organisms for the creation of inorganic nanoscale particles is a potential new development in the realm of biotechnology. An essential milestone in the realm of nanotechnology is the process of creating dependable and environmentally acceptable metallic nanoparticles. Due to its increasing popularity and ease, use of ambient biological resources is quickly becoming more significant in this field of study. The phrase “green nanotechnology” has gained a lot of attention and refers to a variety of procedures that eliminate or do away with hazardous compounds to repair the environment. Green nanomaterials can be used in a variety of biotechnological sectors such as medicine and biology, as well as in the food and textile industries, wastewater treatment and agriculture field. The construction of an updated level of knowledge with utilization and a study of the ambient biological systems that might support and revolutionize the creation of nanoparticles (NPs) are presented in this article.

## Introduction

Simply said, biotechnology is the use of living organisms and their components in industrial processes and products. It is not an industry in and of itself, but rather a significant technical development that will have a big influence on a wide range of industrial sectors in the future. The study of events that occur in materials at the nanoscale is known as nano-science (Cortez-Jugo et al. 2021). Since biological structures like RNA, DNA, and sub-cellular organelles may be thought of as nanostructures, biology and biochemistry have also been intimately linked to nano-science. A diverse and interdisciplinary discipline, nanotechnology examines numerous areas of nanoscale science and technology (Salem and Fouda 2021; Salem et al. 2023). Nanoparticles, the fundamental units of nanotechnology, have a size between 1 and 100 nm. Due to their fundamental and technological relevance, metal nano-particles including gold, zinc, silver, selenium, and copper have recently attracted a lot of attention (Abdelghany et al. 2022; Soliman et al. 2022b; Salem et al. 2020, 2021; Shehabeldine 2022a, b). Metal sulfide nanoparticles have received a lot of interest due to their remarkable characteristics and prospective uses in electrical, optical, and optoelectronic devices. Well-aligned nanostructure arrays on substrates are very appealing due to their improved characteristics and innovative uses. These nanoparticles are distinct from metallic particles in terms of their catalytic, electrical, and optical properties (Lai et al. 2012). Usually, physical, chemical, and biological processes may be used to create nanoparticles. In the physical and chemical processes, sodium borohydride and other strong-chemical reducing agent are utilized, whereas sodium citrate, alcohols, gamma- and UV radiation, among others, are the weak-chemical reducing agent (Elakraa et al. 2022; Salem et al. 2022a; Abdelmoneim et al. 2022). According to studies, producing nanoparticles via biological techniques is a cheap and environmentally benign process. To date, biological agents including bacteria, fungi, yeast, actinomycetes, and plants have been used to show the production of nanoparticles (Salem and Fouda 2021; Salem et al. 2023). A new and developing environmentally friendly science is the synthesis of nano-materials utilizing bacteria or plants. Many researchers have been synthesizing nano-materials using biological models (Soliman et al. 2022a; Al-Rajhi et al. 2022; Al-Zahrani et al. 2022a, b). The use of science in nanotechnology to manipulate matter at the atomic level. The characteristics of matter are very different from bulk materials at this level. The production, use, and assessment of structures, devices, and system where the shape and size are regulated at the tiny size regime are also included by this term (Dong et al. 2021). In other phrases, the fields of nano-science and nanotechnology concentrate on (i) developing synthetic approaches and surface observational tools for creating materials and structures, (ii) observing the changes in physical and chemical attributes brought about by miniaturization, and (iii) utilizing such features to create new and useful devices and materials. In the fields of solar energy conversion, catalysis, medicine, and treatment of water, nano-materials may offer answers to technical and environmental problems (Ismael 2020; Saied et al. 2021, 2022).

## Methods of synthesis of nano-particles

Numerous procedures have been developed for the synthesis of nano-materials due to the large variety of uses afforded by these materials in various branches of research and industry (Jamkhande et al. 2019). To create nano-particles of various sizes and forms, diverse synthesis techniques employ a variety of chemical, physical, and biological factors (Sharaf et al. 2022; Eman et al. 2022; Salem et al. 2023).

## Physical and chemical techniques

A lot of investigators have discovered several physical as well as chemical methods for achieving the fabrication of nanostructures such as shapes that may be used in a wide range of industries (Ijaz et al. 2020). As unique ways for creating such single geometries in nanostructures, photolithography, ball milling, ion-beam lithography, micro-contact printing, dip-pen lithography, evaporation–condensation, electro-chemical synthesis, and nano-imprint lithography are reflected (Salem et al. 2023). Physical methods are also available to achieve the geometries. Chemical processes, on the other hand, begin with the reduction of metal ions to metal atoms, followed by a managed bulk of atoms (Sotiropoulou et al. 2008). Chemical and physical approaches have generally been used in the production of various types of nanomaterials due to their specificity and generation of monodisperse nanostructures. The chemical reduction process, which involves reducing metal particles to nano-particles using chemical-reducing agents like sodium borohydride or sodium citrate, is the technique that produces nano-particles most often (Panigrahi et al. 2004). Metal, metal oxide and metal sulfide nanostructures have been generated using a variety of methods, including metal ion reduction by various reducing agents such as hydrazine hydrate and sodium-borohydride, solvothermal formation, sol–gel technique, microwave-assisted formation, laser ablation, ion sputtering, gamma-ray irradiation, micro-emulsion, electro-chemical reduction, and autoclaving (Phuoc 2014). The most commonly used processes for nanomaterials synthesis have one or more drawbacks, such as high operating costs, toxicity, and inefficiency, generating various environmental problems. As a consequence, increasing environmental through biological and green synthesis methodologies is strongly advised.

## Green synthesis of NPs

**Fig. 1:**
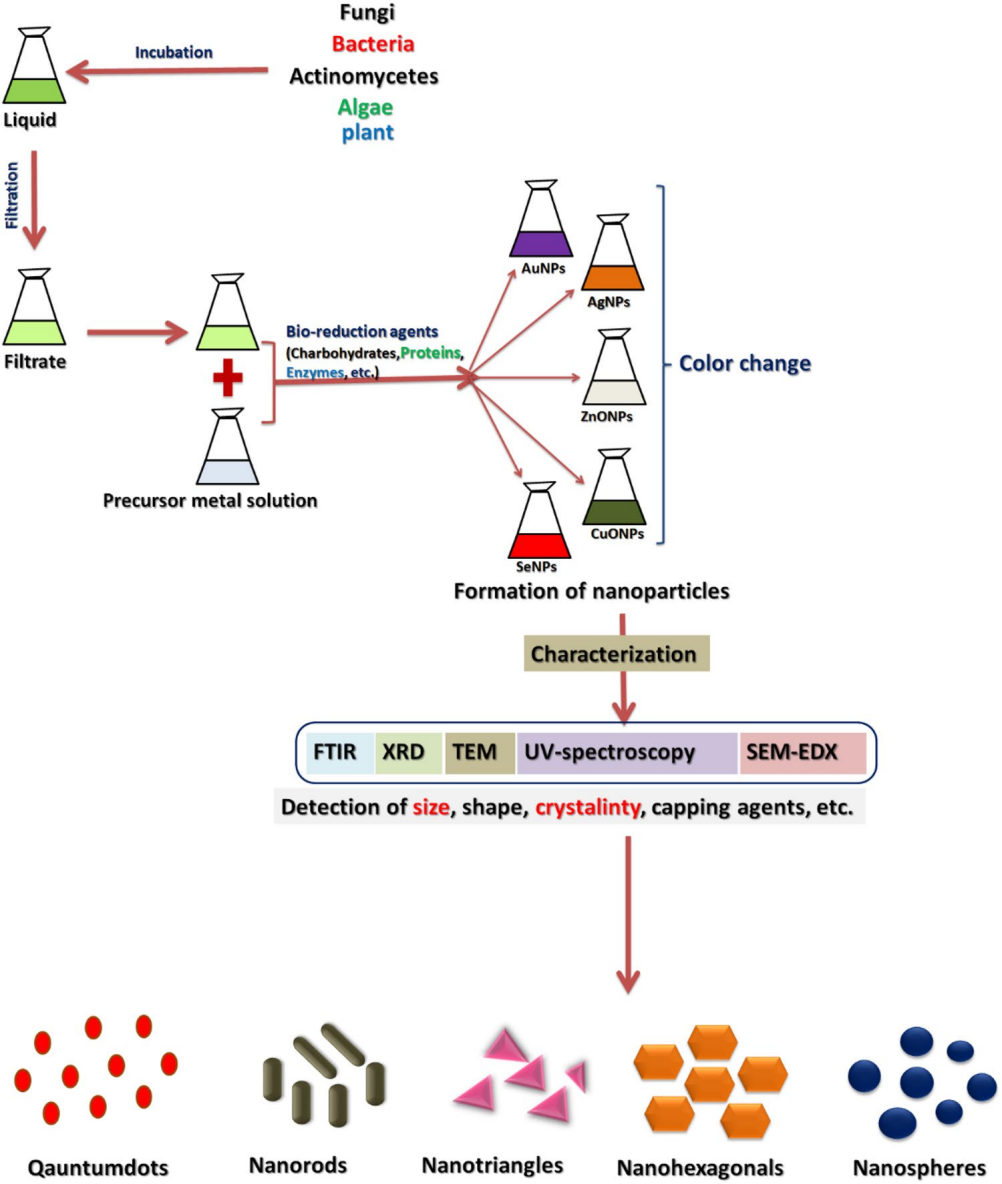
Schematic outlines of the various biological approaches for the formation of nanoparticles (Shaheen et al. 2021a)

Utilizing a variety of species as long-lasting, ecologically secure precursors, biological synthesis can create durable, biofunctional nano-particles. There have been reports of the greener synthesis of NPs using biomass filtrate derived from a variety of biological systems, including yeast, actinomycetes, plant extract, fungi, algae, and bacteria (Alsharif et al. 2020; Salem and Fouda 2021). Recently, different organisms including uni-cellular and multi-cellular are used for bio-synthesis of nano-particles as represented in Fig. 1. The synthesis process of NPs may be viewed as a bottom-up process in which biomolecules released by the organism, including such enzymes, proteins, polysaccharides, and carbohydrates, oxidize or reduce metallic ions to generate NPs (Salem et al. 2023). Because different types of microbes interact with metallic ions in different ways, a thorough understanding of the process behind the synthesis of microbial NPs has not yet been achieved. The size, morphology, and form of the biosynthesized NPs are ultimately influenced by the biochemical metabolism, interactions, pH and temperature effects of a particular microorganism. Nanoparticles are formed either intra-cellular or extra-cellular depending on microorganisms type. Researchers have made use of cell extracts for the biological production of NPs (Saied et al. 2021; Salem et al. 2022b, c; Suba et al. 2021).

### Algae fabrication of NPs

Algae are saltwater microorganisms that have been shown to not only absorb heavy metals from their surroundings but also to create metallic nanoparticles (Uzair et al. 2020). Algae could potentially have a significant economic impact in the future if cost-effective both downstream and upstream processing is developed. Algae are well-known for their ability to collect ions of heavy metals and restructure them into more flexible forms (Nowicka 2022). Depending on the algae species and method of activity, nanomaterials can be synthesised extra- or intra-cellularly. The production of nanomaterials from a broad variety of algal substances has proven to rank among the most current and novel fields of biochemical study due to their ability to reduce metal ions (El-Refaey and Salem 2023). Among the various algae, the most extensively studied algae for the biosynthesis of NPs are brown, red, blue-green, micro and macro green algae (Chaudhary et al. 2020). For example, dried *Chlorella vulgaris* microalgae cells were grown to make Au-NPs using reducing tetra-chloroaurate ions to generate gold-NPs (Luangpipat et al. 2011). Brayner et al. demonstrated the use of cyanobacteria in the production of gold, platinum, silver, and palladium NPs (Brayner et al. 2007). *Jania rubins*, *Pterocladia capillacae*, *Colpmenia sinusa*, and *Ulva faciataand* were the four marine macro-algae utilized for NP production (El-Rafie et al. 2013; Azizi et al. 2013; Rajeshkumar et al. 2014).

### Fungi and yeasts fabrication of NPs

Fungi are the largest group among microbes, which are used in multiple applications in different sciences such as bioremediation, enzyme production, and nanotechnology (Selim et al. 2021; Ahmed et al. 2022; Mohamed et al. 2019). Fungi have sparked a lot of interest in manufacturing metallic nanoparticles since they have several benefits over bacteria when it comes to nanoparticle synthesis. The simplicity of scaling up and downstream processing, as well as the economic feasibility and the existence of mycelia, which provides a larger surface area, are all significant benefit (Shaheen et al. 2021a). A biomineralization mechanism is used in fungal-based nano-materials production, which involves internal and extracellular enzymes and biomolecules reducing various metal ions (Spagnoletti et al. 2019). In addition, Au, Ti, Se, Cu and Zn have been identified as the next most important metal ions employed by fungus in the production of NPs. More research on nano-materials biosynthesis has been done on *Fusarium*, *Aspergillus*, *Trichoderma*, *Verticillium*, *Rhizopus*, and *Penicillium* species (Salem and Fouda 2021). *Fusarium oxysporum* can produce zinc sulfide (ZnS), lead sulfide (PbS), Cadmium sulfide (CdS), and molybdenum sulfide (MoS) nano-materials, When the right salt is given to the growing media (Ahmad et al. 2002). For illustration, Mohamed et al. (2019) established the fusion of ZnO NPs by using *A. niger* and *F. keratoplasticum* that ensued in the development of ZnONP with the steady diameter size of 8–38 nm and 10–42 nm for hexagonal and nanorod ZnO-NPs, respectively, and high mono-dispersity elements (uniformly distributed) deprived of any agglomeration. Besides, the authors recommended that the protein buried by the fungi was bound and reduced the orbicular ZnONPs and prohibited the NP from agglomerate. In adding, filtrate-cell free (FCF) of *Aspergillus terreus* substitute was utilized in the amalgamation of ZnONP. On that version, the FCF was patented to create NP after the snow of zinc acetate elucidation with the size of 10–45 nm. In addition, the FTIR-spectra study validated the existence of proteins and surplus biological compounds in the ZnONP formed. The results show how the produced ZnO-NPs were analysed using various tools including TEM, FTIR, TGA, and XRD analyses (Fouda et al. 2018). *Aspergillus terreus* at ambient temperature was used for the amalgamation of CuO NPs, which were partitioned for eliminating copper from combined circuits and allowed to exist in nano form. CuO NPs of an average size of 11–47 nm were assembled extracellularly by biomass of fungal cell isolated from a soil sample in Egypt, and infrared spectroscopy [IR] analysis revealed that amide groups within proteins were responsible for the consistency and coating agents surrounding the CuO NPs. CuO NPs confirmed by other techniques (Shaheen et al. 2021b). The extracellular synthesis of nanoparticles in huge quantities, with straightforward downstream processing. Different processes used by yeast strains of different genera for nanoparticle formation result in significant differences in size, mono dispersity, particle position, and characteristics (Lian et al. 2019). These molecules determine the mechanism for the formation of nanoparticles and stabilize the complexes in the majority of the yeast species studied. Resistance is defined as the ability of a yeast cell to convert absorbed metal ions into complex polymer compounds that are not toxic to the cell. In the mass production of metal nanoparticles, yeast production is easy to manage in laboratory settings, and the rapid growth of yeast strains and the use of basic nutrients have various advantages(Salem 2022a). *Candida glabrata* and *Saccharomyces pombe* yeast strains have been described for the production of intracellular synthetized silver, titanium, cadmium sulfide, selenium, and gold nanoparticles for this purpose(Soliman et al. 2018; Boroumand Moghaddam et al. 2015). The extra cellular bio-formation of SeNP was performed by using yeast (*Saccharomyces cerevisiae*) extract. Production of Se NPs is confirmed by the absorption peak at 300 nm in UV–Vis spectroscopy due to the surface Plasmon resonance of Se NPs. It is also characterized by FT-IR and XRD. The Se NPs around 5–51 nm were formed (Salem 2022b).

### Plants fabrication of NPs

Plant sections such as leaves, stems, roots, shoots, flowers, barks, seeds, and their metabolites have all been effectively utilized in the fabrication of nanoparticles (Abdelghany et al. 2022; Al-Zahrani et al. 2022a; Aref and Salem 2020). Green synthesis of NPs using plants has more benefits than other approaches since it is a one-step procedure, economical, produces a large quantity of metabolites, is cost-effective, and ecologically sound (Ahn et al. 2019). Using plant extracts such as *Cinnamom zeylanicum, Pinus resinosa, Ocimun sanctum, Curcuma longa, Anogeissus latifolia, Glycine max, Musa paradisica, Pulicaria glutinosa, Cinnamomum camphora, Doipyros kaki,* and *Gardenia jasminoides*, green production of nano-materials has been described. Many studies have been published on the formation of nanoparticles through biosynthesis (AuNPs, AgNPs, ZnONPs, FeNPs, etc.) utilising extracts of various plant sections (Salem and Fouda 2021). Hashem and Salem (2022) recognized the synthesis of Se NPs using *Urtica dioica* which formed a pure crystalline and spherical shape of Se NPs with the size ranged from 5 to 43 nm. Production of Se NPs is confirmed by the absorption peak at 300 nm in UV–Vis spectroscopy. In addition, the obtained SeNP was characterized by different tools as SEM–EDX, TGA, FTIR, TEM, and DLS analysis. SeNPs created by biosynthesis were tested for their antimicrobial and anticancer properties.

### Bacteria and actinomycetes fabrication of NPs

Prokaryotes have mostly been studied as a method of producing nanomaterials. Bacteria are a good choice for research because of their prevalence in the environment and ability to adapt to adverse conditions. They are also fast growing, inexpensive to nurture, and simple to handle. Temperature, oxygenation, and incubation time are all easily adjustable growth conditions (Barghoth et al. 2023; Selim et al. 2022; Bakry et al. 2022). Bacteria are known to synthesise inorganic compounds both intra and extracellularly. Outside the cells, *Pseudomonas stutzeri *was utilised to create Ag-NPs. Moreover, numerous Gram-negative and Gram-positive bacterial strains, including *A. calcoaceticus*, *B. amyloliquefaciens*, *B. flexus*, *B. megaterium*, and *S. aureus*, have been employed for both extracellular and intracellular production of AgNPs. These AgNPs come in a variety of shapes, including spherical, disc, cuboidal, hexagonal, and triangular. Bacteria have been proposed as a potential biofactory for the manufacture of NPs such as silver, gold, palladium, platinum, magnetite, titanium, titanium dioxide, cadmium sulfide, selenium, and other nanomaterials. These actinomycetes are capable of producing antibiotics as secondary metabolites. Actinomycetes have been discovered to have an important role in the formation of nanomaterials. Actinomycetes are a kind of microbe that is used in the synthesis nanomaterials.

Actinomycetes generate nanoparticles with great polydispersity and stability, as well as biocidal efficacy against a wide range of illnesses (Manivasagan et al. 2016). Au-NPs have been successfully synthesised by *Thermoactinomycete* sp., *Rhodococcus* sp., *Streptomyces viridogens*, *Nocardia farcinica*, *Streptomyces hygroscopicus*, *Thermomonospora* sp. (Składanowski, et al. 2017). On the other hand, *Streptomyces* sp. was effectively utilized for producing Cu, Ag, and Zn-NPs (Alani et al. 2012; El-Gamal et al. 2018; Hassan et al. 2018, 2019).

## Mechanism of bio-synthesis

**Fig. 2:**
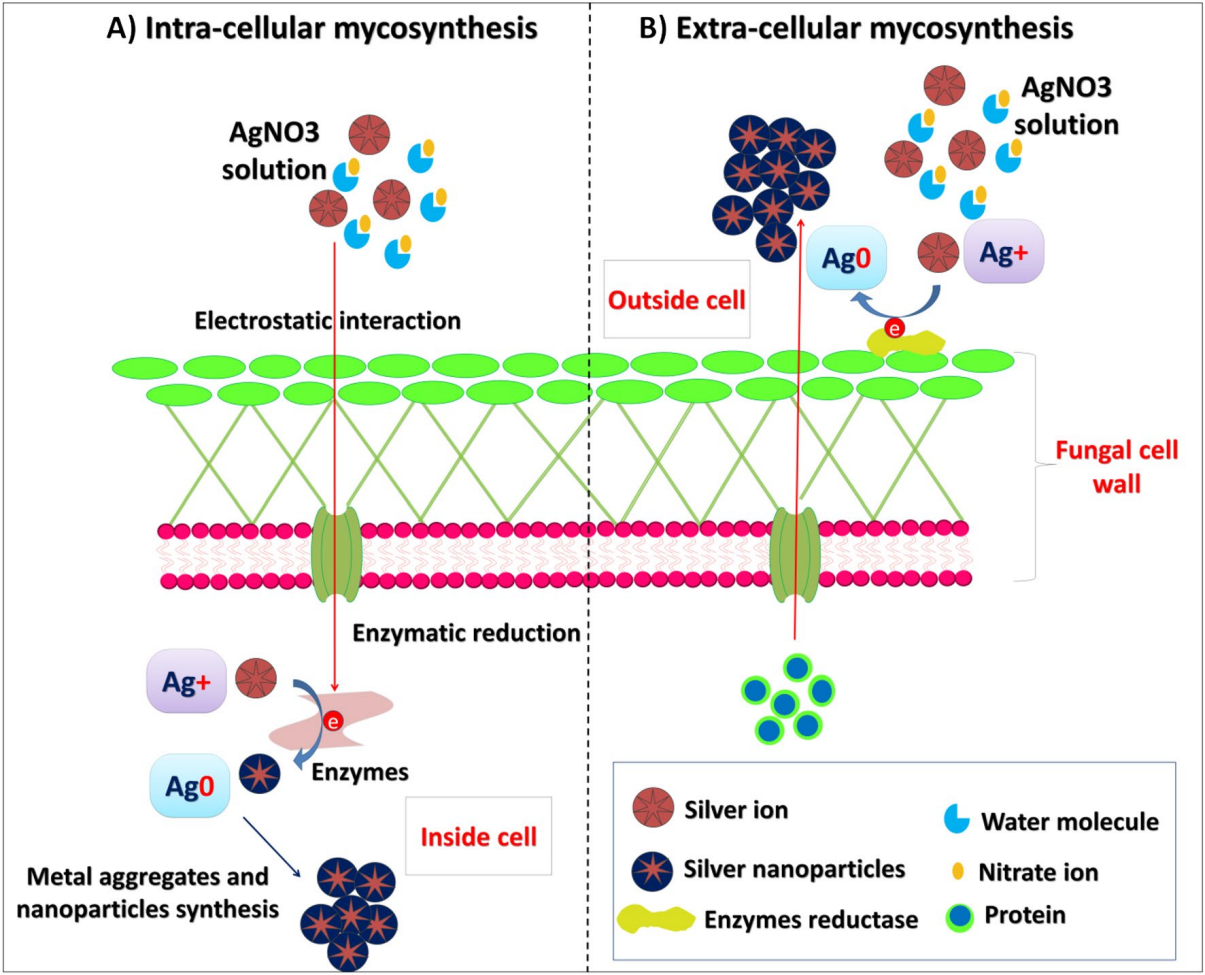
Hypothetical mechanism for NPs biosynthesis (Shaheen et al. 2021a)

The mechanism for intra- and extra-cellular formation of nano-materials is different in numerous biological models. Microorganisms’ cell walls play a significant influence in the intracellular creation of nano-particles. The positively ( +) charged metal ions interact electrostatically with the negatively (−) charged cell wall. The metal ions are bioreduced to nano-particles by the enzymes that are present in the organism cell wall, and then the smaller nano-particles are dispersed out through the cell wall. The exact mechanism for creating nano-particles utilizing biological models has not yet been thought of. This is due to the fact that various biological agents interact with metal-ions and metal oxide in different ways, resulting in the creation of nanoparticles. Metal ions and metal oxide are converted to nanoparticles by the enzymes found within the cell wall, which then diffuse away from the cell membrane. A stepwise mechanism for intra-cellular formation of nano-particles using *Verticillium* sp. (Mukherjee et al. 2001). Explains the mechanism of the biosynthesis of nano-particles involving bio-reduction capping, and trapping. When the cell surface contact with metal ions, it inter-acts electro statically and traps the ions. The cell wall’s enzymes convert metal ions into metal and/or metal oxide nano-particles. The mechanism involves the reduction of Ag^+^ to Ag^0^ via intra-cellular redox value, and proteins (enzymes) which formed by the cell. These reactions may occur either intra-cellular or extra-cellular (Fig. 2) (Shaheen et al. 2021a). Biological molecules, which can be found in plant extracts or released by bacteria and fungus, operate as capping and reductants. These substances are sugar, carbohydrate, enzymes, and proteins that use an oxidation/reduction process to convert metallic ions from (M^+^) to (M^0^). Reduced metallic form aggregates and forms clusters of nanomaterials, which may be validated by changing colour in the reaction mixture (Qamar and Ahmad 2021).

## Advantages of biological synthesis NPs

During the last few years, there have been astonishing advances in the field of bioformed nanostructures and their applications. The bioformation of nanomaterials offers conventional advantages such as care and ecological creation, economic effectiveness, and the biocompatibility of mixed nanomaterials (Singh et al. 2018). The biogenic process of fusion also has the advantage of not requiring an extra step of coating or attaching bioactive facilities or microbe to the nano-particle surface to create constant and pharmacologically active atoms, which is otherwise required in physico-chemical mixtures. In addition, the time required for the biosynthesis of nanoparticles is far less than that required for physicochemical techniques. Several intermediates have developed fast synthesis pathways with excellent nanoparticle yields employing other microbes. Notwithstanding the numerous benefits provided by the biotic pathway for creation, the polydisparity and size of metal oxide nano-particles remain significant and stimulating challenges. In addition, significant effort is chosen to increase amalgamation competency, unit size control, and feature. As a result, several modern intelligences have developed a consistent approach for the biosynthesis of nanoparticles with monodispersity in size and form. Biofilms are a high-potential approach for the effective production of nano-particles and microorganism (Tanzil et al. 2016). Biosynthesized nanoparticles have recently been accepted as the most energetic form of bacterial growth (Iravani and Varma 2020). The incorporation of nanoparticles into microorganisms provides additional benefits, such as high biomass demands and broad external regions, which can lead to greater real and local biosynthesis. Despite extensive research on metal oxide nano-particle fusion in species such as fungus and bacteria, little is known about the calming machinery of nano-particles in biofilms. One of the key constraints in bio-mediated synthesis is the comprehensive and systematic consideration of mechanical structures of nanoparticle biofabrication (Salem 2022b).

## Factors affecting NPs biosynthesis

**Fig. 3:**
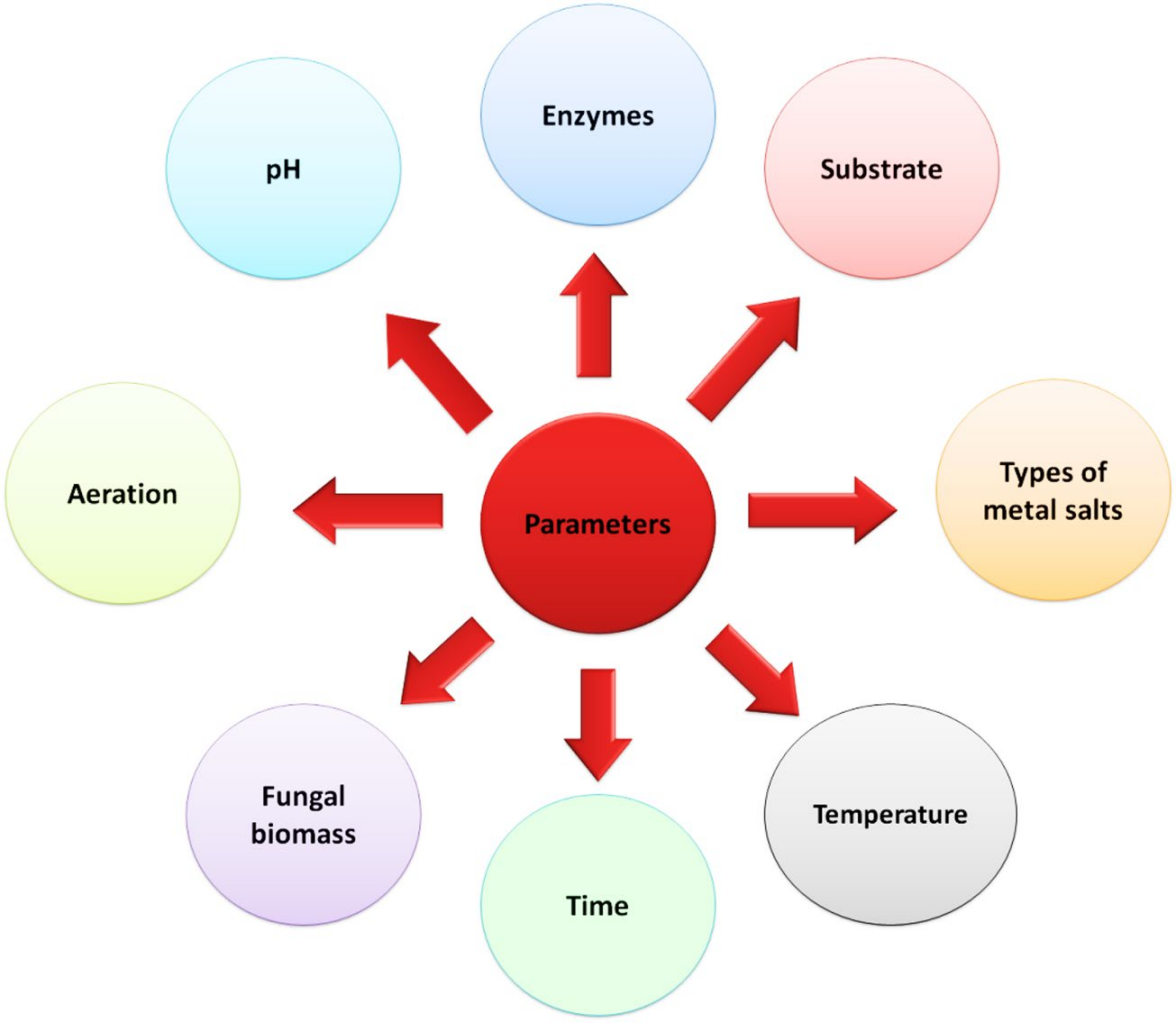
Parameters affecting biosynthesis of nanoparticles (Shaheen et al. 2021a)

There are numerous factors that influence the biosynthesis process for NPs as biomass, temperature, precursor conc. and time contact, pH and the being of a certain enzyme (Fig. 3) (Shaheen et al. 2021a). Adjust the size and shape of metal nano-materials has appeared by either compelling their environmental development or shifting the functional molecules. Aeration, pH, incubation time, redox conditions, temperature, mixing ratio, salt content, and irradiation have all been looked into as ways to improve the reaction conditions for the biogenesis of NPs. The physical and chemical parameters affect the size and form of NPs. The ability to identify the size and structure of nanoparticles depends heavily on the reaction mixture’s optimal metal ions concentration, temperature, and pH. Planning variables including substrate concentration, pH, temperature, and exposure time to substrate may, to some extent, impact the pace of intra-cellular nanoparticle formation and later the size of the NPs. Researchers investigated the optimization of numerous parameters as temperature, inoculum of biomass, pH and precursor conc. in the mycosynthesis of AgNP, ZnONP and AuNP (Al-Kordy et al. 2021; Thakral et al. 2021; Soleimani et al. 2022; Koçer and Özçimen 2022; Desai et al. 2021; Shirzadi-Ahodashti et al. 2022).

## Stabilization and characterization of nano-particles

Nanostructured metal colloids have been obtained by “top-down” and “bottomup” approaches. It has been published that when gold and silver nano-particles formed then they are stabilized by the proteins. Proteins can bind to gold nano-particles either through free-amine (NH) groups or others in the proteins (Pourali et al. 2018; Bhambure et al. 2009). One or more of these proteins may be enzymes that reduce chloroaurate ions and cap the gold nano-particles formed by the reduction process. So a separate protein affects the gold nanoparticles’ stability and capping. Tetrachloroauric acid is reduced by various reducing agents in the most common process of creating AuNP. However, gold nanoparticles are highly reactive due to their high surface energy. A unique stabiliser must be utilised to prevent their accumulation or precipitation. Biomolecule monolayer is one of the often used techniques for passivating the surface of AuNP. Recently, homopolymers and block polymers that can successfully stabilise AuNP through steric stabilisation have demonstrated great promise for use in innovative materials (Doghish et al. 2022). It is important to keep in mind that polymer chains adsorbing on the surface of Au NPs can both improve the stability of the gold cores and functionalize them, especially when intelligent polymers are utilized (Hashem et al. 2022a). Additionally, stabilizing substances that are either adsorbed or chemically attached to the surface of the Au NPs are needed. These stabilizing substances, which are frequently also referred to as surfactants, are usually charged, causing the identically charged NPs to resist one another and become colloidally stable. A wide range of stabilizers can be used to stabilize Au NPs (ligands, polymers, surfactants, dendrimers, bio-molecules, etc.) (Marouzi et al. 2021). The physico-chemical characterisation of the produced nanoparticles is a crucial step in the biosynthesis of nanoparticles. Understanding properties like size, shape, surface area, homogeneity, and others can help one better understand how to regulate the synthesis of nanoparticles for use in industry. Different methodologies may be used to characterise the nano-materials, which is crucial for understanding their diverse physio-chemical properties (Ibrahim et al. 2021a). The following categories cover the most often used tools:

1. X-Ray diffraction (XRD).
2. Scanning electron microscopic (SEM).
3. Transmission electron microscopic (TEM).
4. Ultraviolet–visible (UV–Vis) spectroscopic.
5. Fourier transform-infrared (FTIR) spectroscopic.
6. X-ray photoelectron spectroscopic (XPS).
7. Atomic absorption spectroscopic (AAS).

The structural features are crucial in studying the content and behavior of bonding materials. It gives a variety of information regarding the subject material’s bulk qualities. The most frequent methods used to explore the structural characteristics of nanomaterials include XRD, energy dispersive X-ray (EDX), FTIR, XPS, and Zieta size analyzer (Fouda et al. 2020). One of the most essential techniques for revealing the structural features of nanomaterials is XRD. It contains sufficient information on the crystallinity and phase of nanomaterials. It also offers an approximate estimate of particle size using the Debye Scherer calculation. XPS is commonly used to establish the actual elemental ratio and bonding type of the elements in NPs materials. It is a surface-sensitive approach that may be utilised in-depth profiling research to determine the truly powerful and the compositional change with depth. Well-known optical tools for studying the optical characteristics of nanomaterials include UV–Vis. Morphological aspects of nanomaterials are constantly of significant interest since morphology determines the majority of the attributes of the nanomaterials. There are several morphological characterisation techniques, but microscopic strategies such as TEM and SEM are the most relevant (Salem and Fouda 2021). SEM is a technology that uses electron scanning to offer all relevant details on nanostructures at the nano scale. There is a large body of research in which individuals utilized this approach to investigate not just the shape of their nanostructures, but also the distribution of nanomaterials in bulk or composite. Similarly, considering TEM is grounded in the idea of electron transmittance, it may offer information about the bulk material at low to various magnifications. This approach is used to investigate the various morphologies of Nanomaterials (Das 2020).

## Application of nanoparticles

**Fig. 4:**
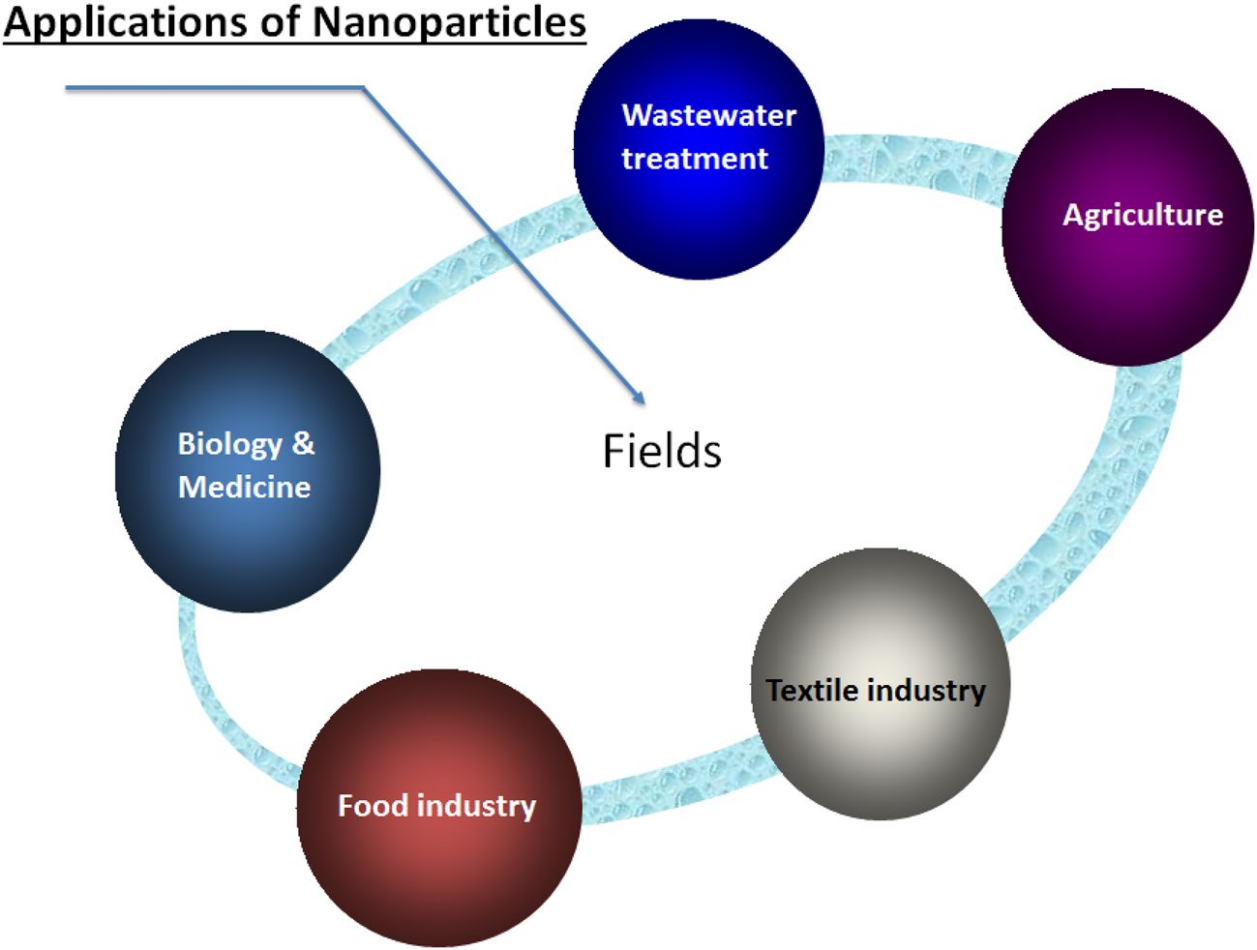
Applications of nanotechnology

Today, nanotechnology is considered an important factor that influences science and industry. Experts claim that nanotechnology has an impact on nations’ economics and future prospects (Fajardo et al. 2022). Through the application of nanotechnology and the bio-production of nanoparticles, there is now renewed optimism for finding solutions to human issues (Dong et al. 2021; Hagde et al. 2022). Fields of application of nano-particles are represented in Fig. 4.

The usage of nano-materials has grown quickly in a variety of industries, including the pharmaceutical and medical sectors, which has prompted the release of new and useful goods on the market (Mughal et al. 2021; Rabiee et al. 2021). Due to the rise and spread of medication resistance among pathogenic microorganisms, various issues have been encountered nowadays when treating them (Shen et al. 2021). The capacity of humans to cure major infectious illnesses is threatened by bacteria's rising rates of antibiotic resistance (Mubeen et al. 2021).

### In medical and biology

The emergence of drug-resistant microorganisms poses a significant problem for medical practitioners, and innovative medications are increasingly being sought to treat a variety of ailments. Significant breakthroughs in nano-medicine enable NPs-based bio-imaging and early diagnosis systems, as well as the treatment and diagnosis of illnesses caused by drug-resistant microorganisms. Nanotechnology offers novel solutions for making elemental Se safe, absorbable, and bioavailable for aquatic organisms in the form of Se nanoparticles (Ferro et al. 2021; Hashem et al. 2022b). This is because of the tiny size and large surface area of the element. Selenium nano-particles (Se NPs) have shown outstanding bioavailability, with a low toxicity level, and can exert an envisioned activity at much lower concentrations than organic or in-organic Se compounds (Menon et al. 2018). Further, SeNPs exhibit biological activities that promote optimum substitutions for various seleno compounds (Yanhua et al. 2016). Extra-cellular biogenesis of SeNP by *Penicillium corylophilum* has been characterised to be of poly-dispersed nature (Salem et al. 2021). It has been discovered that a cell-free extract of *Penicillium expansum* has a high potential for producing Se nanoparticles, and that the process can be regulated to alter SeNP’s structure. SeNPs with a size range of 4–12.7 nm have only been found to be spherical (Abu-Elghait et al. 2021). The extra cellular bio-formation of SeNP was performed by using yeast (*Saccharomyces cerevisiae*) extract. Production of Se NPs is confirmed by the absorption peak at 300 nm in UV–Vis spectroscopy due to the surface Plasmon resonance of Se NPs. It is also characterized by FT-IR and XRD. The Se NPs around 5–51 nm were formed. The effectiveness of biosynthesized Se NPs as antimicrobial agents against food-borne pathogens was evaluated. With minimum inhibitory concentrations (MIC) of 62.5 µg/mL, 125 µg/mL, 250 µg/mL, and 500 µg/mL against *S. aureus*, *E. coli*, *A. fumigatus*, and *A. niger*, respectively, Se NPs demonstrated potential antibacterial efficacy against food-borne pathogens. Finally, a mechanism was designed for the effect of nanoparticles on microbes (Hashem et al. 2021; Al-Rajhi et al. 2022). In addition, Mohamed et al. (2021) have proven an extra-cellular formation of ZnONP and CuO NPs using *P. chrysogenum*. Also, the UV–vis spectra results detailed strong peaks at 380 nm and 335 nm, respectively, implying the positive realization of ZnONP and CuO NPs, individually. The produced ZnO and CuONPs were analyzed using various methods including TEM, SEM, FTIR, and XRD analyses. The effect of ZnONP and CuO NPs on pathogenic bacteria was also investigated in this study since it demonstrated the potency of ZnONP and CuO NPs against these microbes. They also investigated how well particles prevented the formation of biofilms; the outcome was dependent on the particle concentration, and encouraging results were demonstrated as potential medicinal uses. Among metallic nanoparticles, Se NPs have attracted the attention of researchers and have special properties such as stability in environmental conditions and synthesis at low temperatures (Wadhwani et al. 2016). In general, there are numerous approaches for the biosynthesis of SeNP, one of these methods is the plant-mediated method that is, the synthesis of nano-particles using plant (Hashem and Salem 2022). Hashem and Salem (2022) recognized the synthesis of Se NPs using *Urtica dioica* that formed a pure crystalline and spherical shape of Se NPs with the size ranged from 5 to 43 nm. Production of Se NPs is confirmed by the absorption peak at 300 nm in UV–Vis spectroscopy. In addition, the obtained SeNP was characterized by different tools as SEM–EDX, TGA, FTIR, TEM, and DLS analysis. SeNPs created by biosynthesis were tested for their antimicrobial and anticancer properties. Results showed that SeNPs showed potential antibacterial efficacy against unicellular and multicellular fungus, as well as Gram-positive [*B. subtilis*, and *S. aureus*] and Gram-negative [*E. coli*, *P. aeruginosa*] bacteria. The Vero normal cell line (CCL-81) was used to test the cytotoxicity of SeNPs, and the IC_50_ value was 173.2. Abu-Elghait et al. (2021) synthesized selenium nano-composite and investigated its anti-biofilm effects against *P. aeruginosa* and *S. aureus* strains. The results showed that this myco-synthesized novel selenium nano-composite can inhibit biofilm and have anti-biofilm effects. Abdelghany et al. (2022) recognized the synthesis of NPs using *Salix tetrasperma* that formed a pure crystal and spherical-shape of ZnONP with a size range of 5–43 nm. Other approaches used to characterise the produced ZnO NP, including SEM–EDX, TEM, and DLS analyses. The disc diffusion technique was used to evaluate ZnONPs’ antibacterial effectiveness. Using the MTT technique to measure cytotoxicity against prostate cancer cells (Pc3). ZnONPs showed good antibacterial action against *S. typhimurium, E. coli, K. pneumonia, B. subtilis, and S. aureus*, with inhibition zones of 28.1, 23.83, 28.33, 23.83, and 33.83 mm, respectively. Inhibition zone of 23.67 mm also inhibited *C. albicans*, however, ZnONPs had no effect on *M. circinelloide* or *A. fumigatus*. ZnONPs showed good antioxidant activity, with an IC50 of 8.73 g/mL as opposed to the plant extract’s IC_50_ of 15.91 mg/mL. The results of a cytotoxicity test showed that ZnONPs were active against Pc3. The success of nanotechnologies in drug delivery can be attributed to improved in vivo distribution, evasion of the reticuloendothelial system and favorable pharmacokinetics (Ferreira et al. 2021). Many forms of NP-sized drug delivery vehicles, such as polymeric micelles, liposomes, dendrimers, and inorganic NPs, have been investigated in cancer treatment to decrease anticancer medication side effects and increase antitumor treatment effectiveness target therapies (González-Ballesteros et al. 2019; Liang et al. 2020).

### In agricultural

Agriculture is the most common application for biogenic nanomaterials. Nanopesticides and nanofertilizers have been extensively investigated and used in agriculture (Badawy et al. 2021; Hashem et al. 2022c). The use of nanoparticles in agriculture will likely increase in the future. A better knowledge of the biochemical, physiological, molecular, and stress tolerance mechanisms of nanomaterials in plants leads to improved plant growth and harvests under stressful situations. The application of nano-genomics-based technology in plant breeding can be used to deploy nanomaterials as transporters of DNA or RNA in plant cells that divert the genes to the target location at a cellular level for gene expression (Shandilya and Tarwadi 2021; Wani and Kothari 2018**).** The use of nano fertilizers improves crop yield and quality with higher nutrient efficiency while simultaneously reducing spillage in the environment and the cost of production, thus contributing to sustainable agriculture. For example, the application of phosphatic nano fertilizers has been found to increase the growth rate by (32%) and seed yield by (20%) of soybean (Glycine max) as compared to those treated with conventional fertilizers. In addition, carbon nanoparticle nanotubes of Au, SiO_2_, ZnO, and TiO_2_ ameliorate the development of plants by enhancing elemental uptake and use of nutrients (Fraceto et al. 2016). A recent study on different crops has also shown increased germination, seedling growth, physiological activity like photosynthetic activity and nitrogen metabolism, m-RNA expression, and some positive changes in gene expression, fostering their potential use in crop improvement (Liu et al. 2021; Salem and Husen 2022). A previous study used *Penicillium expansum* to produce NPs, which presented the possible for the extracellular fusion of ZnO NP with a size range of 3.5–67.3 nm, while they used DLS and SEM–EDX to determine the size and elemental composition. In this study, ZnO NPs were utilized in place of synthetic fungicides to suppress *F. oxysporum*, a harmful phytopathogenic fungus that causes the wilt-disease in *S. melongena* L. (Abdelaziz et al. 2022). To improve the germination of the rain-fed crop, researchers are working on metal oxide nanoparticles and carbon nanotubes. It is also found that carbo nano tubes can enhance the germination of tomato seeds through the better conveyance of moisture. The data shows that carbon nanotubes (CNTs) act as a new pore and facilitate the passage of water by penetrating the seed coat and acting as a way to channelize the water from the substrate into the seeds. Hence, it can enhance germination in the rain-fed agricultural system (Salem and Husen 2022).

### In food

Nano-materials uses in the food sector include Nano particulate delivery methods, manufacturing, food hygiene, and protection. Nanotechnology will undoubtedly bring distinct characteristics in two primary fields of food manufacturing, packaged food, and food functional ingredients in the near future (Ghebretatios et al. 2021). Specific nano-metal oxides, such as ZnO-NPs, were added to polymeric substances utilized in creating packaging sheets to increase their antibacterial capabilities (Espitia et al. 2012). The nanoparticles were incorporated into packing processes while keeping food safe from contamination. The researchers proposed creating nutritious coverings and containers with ZnO-NPs, which have antibacterial characteristics (Rajamanickam, et al. 2012; Prasad et al. 2014). TiO_2_ has been utilized to provide colour and improve dairy products, processed meals, beverages, toothpaste, seeds, and even pharmaceuticals. It's also used to cover candies. As a consequence, the use of packing containers linked by nanoparticles is crucial and a smart approach for maintaining food fresh for an extended period of time with preventing contamination, and food-borne ways. As an outcome, NPs appear to permeate biofilms more effectively, implying that disinfecting and cleaning operations are carried out appropriately (Huang et al. 2015; Shanthi et al. 2016).

### In wastewater

Lately, the use of nanotechnology in treating wastewater has enabled reasonable water, excellent quality, and treating wastewater mixes with fewer reliance on massive facilities (Uddandarao et al. 2019). Metal oxide-dependent nanoadsorbents have a high affinity for metal ions in polluted wastewater, making them excellent for treating wastewater (Das et al. 2022). Metal oxide nanoparticles such as MgO, MnO_2_, TiO_2_, Al_2_O3, Fe3O_4_/Fe_2_O_3_, and CeO_2_ have been researched for their potential in water treatment by a number of researchers (Saraswat 2022). Iron oxides with a wide surface area have strong adsorption, a significant retardation factor, and resistance desorption, making them ideal for pollution cleanup (Shipley et al. 2011). Scientists have produced many varieties of nanoparticles, nanotubes, and nanocomposites using diverse physicochemical procedures to achieve treating wastewater goals, however, their traditional methods of synthesis include dangerous and flammable chemicals, resulting in secondary contamination. As a result, efforts have been undertaken to design biogenic methods and procedures based on plants and bacteria that are both ecologically safe and cost effective. From an economic viewpoint, nanotechnology allows for the usage of extremely difficult energy efficiency and water management (Jain et al. 2021). Regrettably, due to competition from established wastewater treatment methods, expenditures for this revolutionary nanotechnology should be appropriately accomplished. A variety of metal sulfide photocatalysts, such as CuS, CdS, ZnS, Sb_2_S3, and Bi_2_S3 have been developed for the generation of visible-light reactive photocatalysts and can be utilized in certain applications (Lai et al. 2012). The recent advent of lignin-derived nanomaterials has shown significant potential for treating wastewater. They've been reported to be effective catalytic degraders of nitroarenes and dyes, as well as heavy metal removers.

### In textile

The usage of nanomaterials in textile manufacturing has risen fast, with the use of nanomaterials to textile ingredients aimed at improving final textiles in addition to varied behaviors (Eid et al. 2020; Shaheen et al. 2019). Nanotechnology facilitates the development of sophisticated and multi-functional textiles with several creative uses in health, medicine, design, athletics, army, sophisticated fortification, and transportation (Salem and Fouda 2021). Textiles enhanced with any of these nanomaterials have multiple benefits in wound healing, air purification, medicine delivery, skincare, renewable energy generation, and electrical advantages such as the manufacture of on-body diodes, electronics, and circuits (Shah 2022). Active nanomaterials can be integrated physically or chemically into textiles to manufacture antibacterial textiles. TiO_2_, chitosan, N-Halamine, Ag, Cu_2_O, and other antimicrobial agents have been integrated into textiles for antibacterial activity (Andra et al. 2021; Ibrahim et al. 2021b). UV-blocking and antibacterial characteristics are provided by ZnO NPs (Fouda et al. 2018). TiO_2_ and ZnO are structurally stable and are not toxic when exposed to both UV and hot temperatures. Additionally, Nanomaterials have a high surface area to volume %, which results in a considerable increase in UV-blocking radioactive efficacy as compared to bulk particles (Shaheen et al. 2021a). Textile fabrics are treated with a safe amount of hexagonal and nano-rod ZnONPs to improve their qualities such as antibacterial activity against Gram-positive and Gram-negative bacteria and UV-blocking performance (Mohamed et al. 2019).

## Conclusion

Recently, the rapid growth in information that benefit people and the environment has necessitated significant efforts on the part of scientific institutions to develop green different paths to reduce hazardous materials used during various largest economies such as medication, food, textile products, and manufacturing process. Nanotechnology, being a rapidly emerging sector of technology, has found its way into a variety of businesses due to its favorable properties. Biotechnology, on the other hand, is a fundamental component of most modern enterprises as an environmentally benign green method for the biosynthesis of bioactive constituents. The domains of nano-biotechnology and nanotechnology are currently undergoing intense investigation for different treatment applications. In current years, metal nano-particles have been measured widely for numerous biomedical, bio-remediation and bio-sensor needs because of their strange anti-bacterial, anti-oxidant and optical possessions, and large superficial area-to-volume ratio and higher efficacy. The obviously active complexes secreted by the microorganisms have dual role as concrete groups in reducing and calming agent. The method of biological synthesis is simpler, easier, and does not need any hazardous chemicals.

## Data Availability

The data used to support the findings of this study are available in the article.
